# Budd-Chiari Syndrome Associated With Autoimmune Hemolytic Anemia, Immune Thrombocytopenic Purpura, and Lupus Nephritis: A Case Report

**DOI:** 10.7759/cureus.65160

**Published:** 2024-07-22

**Authors:** Soni K Sah, Archana Maurya, Induni N Weerarathna

**Affiliations:** 1 Nursing, Srimati Radhikabai Meghe Memorial College of Nursing, Datta Meghe Institute of Higher Education and Research, Wardha, IND; 2 Child Health Nursing, Srimati Radhikabai Meghe Memorial College of Nursing, Datta Meghe Institute of Higher Education and Research, Wardha, IND; 3 Biomedical Sciences, School of Allied Health Sciences, Datta Meghe Institute of Higher Education and Research, Wardha, IND

**Keywords:** autoimmune disease, shunt, lupus, budd-chiari syndrome, bone marrow

## Abstract

Budd-Chiari syndrome (BCS) is a scarce but severe condition characterized by the obstruction of the hepatic veins, liver congestion, and consequent damage. This series brings up one unusual presentation of BCS associated with autoimmune hemolytic anemia (AIHA), immune thrombocytopenic purpura (ITP), and lupus nephritis (LN), which collectively complicate the clinical scenario. This is a 19-year-old woman who was admitted for abdominal pain, hepatomegaly, ascites, and jaundice. Her history included the diagnosis of systemic lupus erythematosus. Laboratory findings revealed hemolytic anemia, thrombocytopenia, and impaired renal function. Imaging investigations were done to prove the diagnosis of BCS. The patient's complex autoimmune profile, characterized by the simultaneous presence of AIHA and ITP with LN, underlined the multifaceted nature of her condition. This case underscores the diagnostic and therapeutic challenges posed by the co-existence of BCS with AIHA, ITP, and LN, highlighting the critical role of a multidisciplinary approach in managing such complex cases effectively. Timely diagnosis and targeted treatment strategies are essential for improving outcomes in these patients.

## Introduction

The uncommon condition known as Budd-Chiari syndrome (BCS) is distinguished by the restriction of the hepatic venous outflow at every phase, from the atrio-caval junction to the microscopic hepatic veins. The prevalence of BCS in India can be estimated to range from 0.0001% to 0.001% [1]. One in a million adults has BCS. It is an uncommon illness that, if not treated appropriately, could have disastrous consequences [2]. Hepatomegaly, ascites, and abdominal discomfort are the features of BCS. Clinical signs can range from fulminant liver failure to asymptomatic occurrences [3]. A peritoneovenous shunt (PVS), surgical portosystemic shunt, transjugular intrahepatic portosystemic shunt (TIPS), liver transplantation, or paracentesis can all be used to treat medically refractory ascites, which is a significant problem in BCS [4]. In BCS, liver congestion can cause the spleen to inflate; an enlarged spleen can lead to more significant destruction of red blood cells, contributing to hemolytic anemia [5]. Severe autoimmune anemia can arise as an uncommon complication after BCS, as, in BCS, the functioning of the liver is normal. The key risk factors for BCS include thrombophilic conditions [2]. BCS's etiology is primary, mainly due to venous thrombosis, and secondary to extrinsic compression from tumours or other masses [6]. Here, we present the case of a 19-year-old patient diagnosed with BCS due to venous thrombosis and failure of the shunt.

## Case presentation

Clinical history

**Figure 1:**
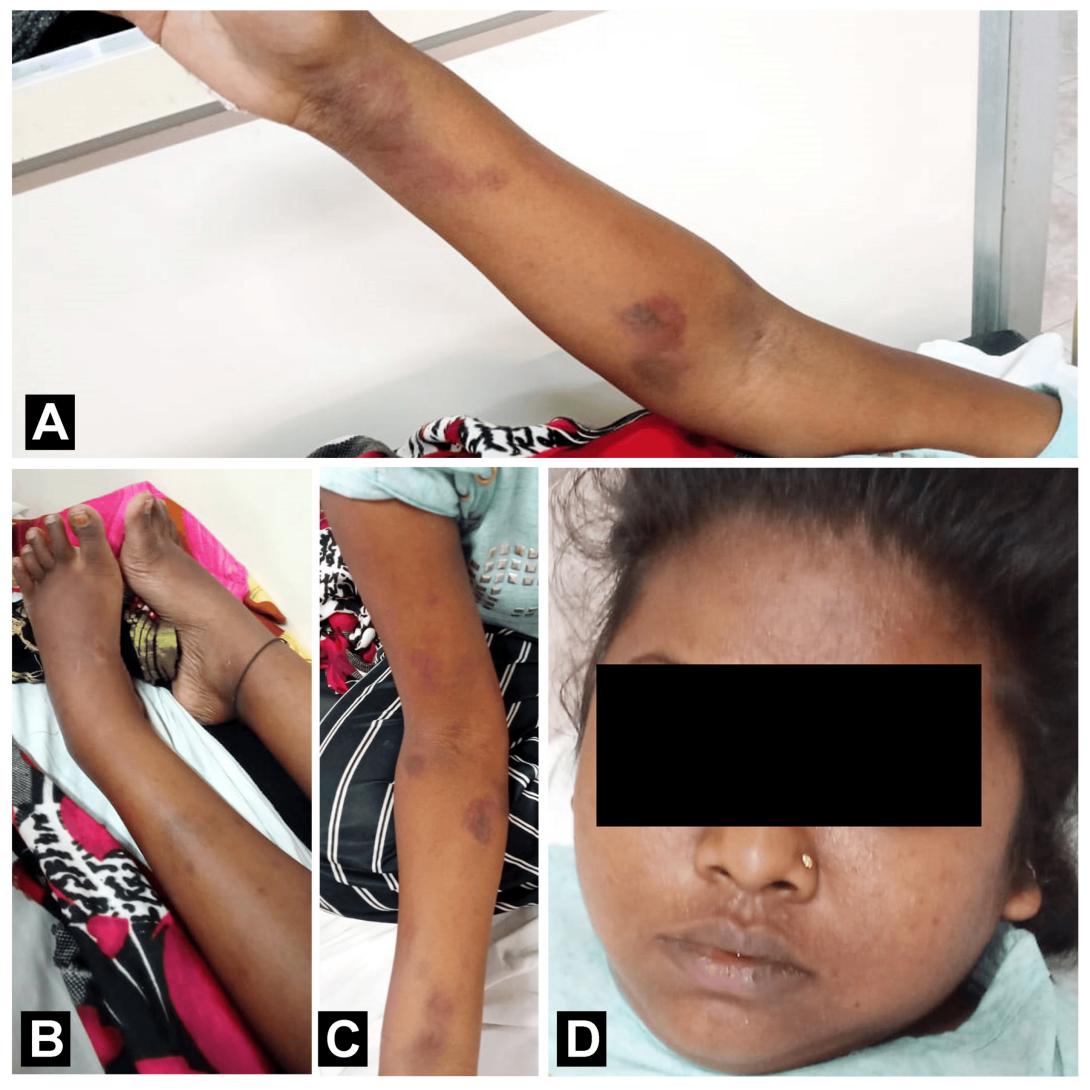
Pre-treatment of the patient: (A) petechiae over the left hand, (B) swelled legs caused by the accumulation of fluids, (C) petechiae over the right hand, and D) puffiness on the face

A 19-year-old female patient was all right two months back when she started complaining of fever, which was intermittent in nature with no specific aggravating and relieving factors. The patient also complained of ghabrahat associated with palpitations, which were sudden in onset and gradually progressive with no specific aggravating or relieving factors. The patient has had a history of persistent fever, epistaxis, oral ulcers, joint pain, pedal oedema, Raynaud's syndrome, facial puffiness, and abdominal pain with ascites for two months in 2024. She did not have any history of hypertension, diabetes mellitus, tuberculosis, or bronchial asthma. According to her past history, she had Chiari syndrome with status post-direct intrahepatic portocaval shunt at the age of 16, and TIPS was successfully used to treat the gross ascites and abdominal distention. Due to the endothelial dysfunction in immune thrombocytopenic purpura (ITP), the symptoms reappeared in the patient, along with lupus erythematosus with cholecystitis. Figure 1 shows the pre-treatment of the patient.

Examination findings

The patient was found to have acute, chronic liver disease and was diagnosed with BCS based on a Doppler ultrasound. The routine blood tests showed elevated leucocyte (white blood cell (WBC)) counts and platelets, resulting in ITP. Petechiae was seen over her hands. The patient went through a bone marrow biopsy and blood transfusion during the treatment. The histopathological examination of bone marrow received a single container labelled as a bone marrow biopsy, a single thread-like, greyish-brown tissue piece measuring 0.8 × 0.3 centimetres. The section from the bone marrow biopsy showed bony trabeculae with no bone marrow material on histopathology. A chest X-ray was done, which showed bilateral pleural effusion (left > right). An electrocardiogram (ECG) showed sinus tachycardia. All routine investigations were done, and reports were attached with the positive status of the antinuclear antibody (ANA3+) and direct Coombs test (DCT). The patient's prognosis was poor; she went through various further investigations, which identified systemic lupus erythrocytosis with systemic lupus nephritis (LN) with cholecystitis.

Laboratory investigations

The patient has gone for several laboratory investigations, such as a complete blood count (CBC), a liver function test (LFT), and a kidney function test (KFT) (Table 1).

**Table 1:** Various laboratory investigations of the patient, including CBC, LFT, and KFT Hb: hemoglobin; RBC: red blood cell; WBC: white blood cell; g/dL: grams per deciliter; dL: deciliter; cells/mcL: cells per microliter; mmol/L: millimoles per liter; mg/dL: milligrams per deciliter; µmol/L: micromoles per liter; CBC: complete blood count; LFT: liver function test; KFT: kidney function test

Test name	Result	Reference range	Remark
CBC investigation			
Hb	8.8	Male: 13.8-17.2 g/dL	Anemia
		Female: 12.1-15.1 g/dL	
Platelets	0.11	150,000-450,000/dL	Thrombocytopenia
RBC	3.17	Male: 4.7-6.1 million cells/mcL	Decrease
		Female: 4.2-5.4 million cells/mcL	
WBC	8100	4,500-11,000 cells/mcL	Decrease
LFT			
Potassium	3.1 millions/L	3.6-5.2 mmol/L	Decrease
Albumin	2.5 g/dL	3.4-5.4 g/dL	Decrease
KFT			
Creatinine	249.1	Male: 0.7-1.3 mg/dL (61.9-114.9 µmol/L)	Increased
		Female: 0.6-1.1 mg/dL (53-97.2 µmol/L)	

Treatment

The patient received distinct therapeutic regimens during her hospitalizations in 2021 and 2024 (Table 2 and Table 3), reflecting the evolving nature of her condition and the tailored medical interventions required. The following tables summarise the treatments administered during these periods.

**Table 2:** Previous treatment during hospitalization in 2021 OD: once a day; BD: twice a day; TDS: three times a day; mg: milligram

Name	Dose	Route
Eltrombopag 150 mg	OD	Oral
Azoran 50 mg	OD	Oral
Omnacortil 20 mg	BD	Oral
Nicardia 10 mg	TDS	Oral

**Table 3:** Treatment during hospitalization in 2024 OD: once a day; BD: twice a day; TDS: three times a day; mg: milligram; IV: intravenous

Name	Dose	Route
Enoxaparin injection I.P 40 mg	OD	IV
Frusemide I.P diuretics	OD	IV
Rifaximin	BD	Oral
Ursodeoxycholic acid I.P	BD	Oral
Azathioprine 50 mg	OD	Oral
Methylprednisolone sodium succinate	OD	Oral

Follow-up

**Figure 2:**
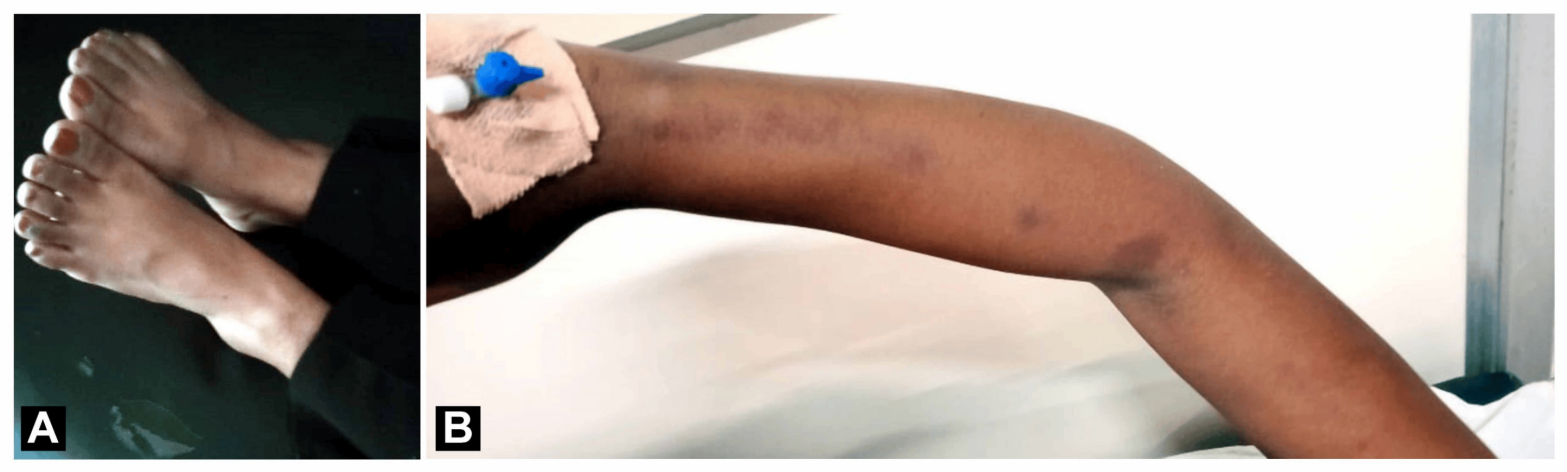
Post-treatment of the patient: (A) decrease swelling of the legs and (B) left hand

The patient's clinical courses were closely monitored throughout the hospitalization, particularly to resolve symptoms and stabilise haematological parameters. Following a comprehensive treatment regimen, the patient exhibited significant improvement and was subsequently discharged, with recommendations for outpatient follow-up. Figure 2 depicts the post-treatment status of the patient.

## Discussion

A significant portion of BCS instances still have an unidentified cause. Factor V Leiden mutation, hyperhomocysteinemia, antiphospholipid syndrome, myeloproliferative neoplasm, celiac disease, and unidentified or inadequately documented etiology were revealed to be the causes of the remaining individuals in a group of 163 instances [7]. This case report describes a 19-year-old female presenting with BCS along with autoimmune hemolytic anemia (AIHA) with ITP and LN with cholecystitis. BCS is a rare disorder characterized by the obstruction of hepatic venous outflow (it could occur due to stenosis or thrombosis), leading to liver cancer [3]. The clinical presentation in our patient aligns with the classical symptoms reported in the literature, including abdominal pain with ascites, fever (on and off), facial puffiness, pedal oedema, and petechiae over bodies. Diagnostic parameters include CBC, liver and renal function tests, lactate, ANA, and Doppler ultrasound [6]. A review of various literature reveals few documented cases of patients simultaneously diagnosed with BCS, AIHA, ITP, and LN; each of these conditions has been individually associated with systemic lupus erythematosus, but their concurrent manifestation in a single person is exceptional. 

Diagnosing this patient posed significant challenges due to the overlapping of clinical manifestations. Treating BCS aims to reduce blockage, stop the clot from getting worse, limit liver damage that worsens over time, and avoid or manage consequences [8]. The treatment strategy involved anticoagulation for the BCS, corticosteroids, and immunosuppressants for AIHA and LN [9]. The Asian Pacific Association for the Study of the Liver (APASL) consensus guidelines state that vitamin K antagonists are preferable over direct-acting anticoagulants when it comes to anticoagulants as first-line therapy [10]. Anticoagulation should be started in all BCS patients with a visible hypercoagulable state and no contraindications. It is the cornerstone of treatment for all cases of BCS [11]. One interventional radiology procedure that can be utilized to reduce portal hypertension and relieve related issues is TIPS [12]. The patient responds favourably to all-encompassing care, and her health has significantly improved. 

## Conclusions

In addition to complicating and making the clinical condition very difficult, the presence of BCS is linked to ITP, LN, and AIHA. This emphasizes the necessity of a multidisciplinary approach and a comprehensive diagnostic workup for treating patients with overlapping autoimmune diseases. Early detection and timely intervention are crucial to enhance the prognosis and prevent serious consequences. This case underscores the importance of increased clinical vigilance and the integration of specialized care to effectively address the complex nature of interconnected disorders. More research and case studies are necessary to better understand these interactions and to develop optimized treatment plans for improved patient outcomes.

## References

[1] Gioia S, De Santis E, Cerbelli B (2020). Small hepatic veins Budd-Chiari syndrome and paroxysmal nocturnal hemoglobinuria - the association of two rare entities: a case report. Pathologica.

[2] Bhatt P, Gupta DK, Agrawal D, Deshmukh H, Rathod K, Shukla A (2019). Budd-Chiari syndrome with spontaneous intrahepatic portosystemic shunts: a case series. J Clin Exp Hepatol.

[3] Aydinli M, Bayraktar Y (2007). Budd-Chiari syndrome: etiology, pathogenesis and diagnosis. World J Gastroenterol.

[4] Kogiso T, Hashimoto E, Ito T (2016). Successful treatment of ascites using a Denver® peritoneovenous shunt in a patient with paroxysmal nocturnal hemoglobinuria and Budd-Chiari syndrome. Intern Med.

[5] Okazaki M, Higuchi T, Koyamada R, Okada S, Fujita Y, Kawasaki T (2015). Coombs-negative autoimmune hemolytic anemia associated with liver cirrhosis due to hepatitis c virus. J Hematol.

[6] AlGhasham N, Abulkhair Y, Khalil S (2015). Flow cytometry screening for paroxysmal nocturnal hemoglobinuria: a single-center experience in Saudi Arabia. Cytometry B Clin Cytom.

[7] Stanciugelu A, Petrică A, Chiriac SD, Iurciuc M, Boruga MV, Balica N, Mederle OA (2022). A rare encounter with two cases of Budd‑Chiari syndrome in the emergency department: a case report. Exp Ther Med.

[8] Hitawala AA, Gupta V (2023). Budd-Chiari syndrome. StatPearls.

[9] Kocheril AP, Vettiyil GI, George AS, Shah S, Geevar T, Dave RG, T SK (2024). Pediatric systemic lupus erythematosus with lupus anticoagulant hypoprothrombinemia syndrome-a case series with review of literature. Lupus.

[10] Solela G, Daba M (2023). Budd-Chiari syndrome as an initial presentation of systemic lupus erythematosus associated with antiphospholipid syndrome: a case report with review of the literature. Open Access Rheumatol.

[11] Torun ES, Erciyestepe M, Yalçınkaya Y (2022). A case of Budd-Chiari syndrome associated with antiphospholipid syndrome treated successfully by transjugular intrahepatic portosystemic shunt. Clin Med Insights Case Rep.

[12] Masek J, Fejfar T, Frankova S (2023). Transjugular intrahepatic portosystemic shunt in liver transplant recipients: outcomes in six adult patients. Vasc Endovascular Surg.

